# Age-Adjusted CKPT App Profiling and Mobility Profiles in Community-Dwelling Older Adults with Near-Ceiling MMSE Scores: A Cross-Sectional Study

**DOI:** 10.3390/healthcare14131868

**Published:** 2026-06-26

**Authors:** Akio Goda, Hideki Nakano, Yuki Kikuchi, Tsuyoshi Katsurasako, Kohei Mori, Atsuko Kubo, Kayoko Nonaka, Shoya Fujikawa, Kohei Iwamoto, Nozomi Mitsumaru, Takaki Shimura, Shin Murata

**Affiliations:** 1 Hokuriku University Well-Being Research Team, Department of Physical Therapy, Faculty of Health and Medical Science, Hokuriku University, Kanazawa 920-1154, Japan; 2 Department of Physical Therapy, Faculty of Health Sciences, Kyoto Tachibana University, Kyoto 607-8175, Japan; 3 Department of Physical Therapy, Faculty of Rehabilitation, Morinomiya University of Medical Sciences, Osaka 559-8611, Japan; 4 Faculty of Allied Health Sciences, Kansai University of Welfare Sciences, Kashiwara 582-0026, Japan; 5 Faculty of Rehabilitation, Department of Rehabilitation, Nishikyushu University, Kanzaki 842-8585, Japan; 6 Department of Physical Therapy, Faculty of Rehabilitation, Reiwa Health Sciences University, Fukuoka 811-0213, Japan; 7 Graduate School of Health Sciences, Kyoto Tachibana University, Kyoto 607-8175, Japan; 8 Department of Rehabilitation, Kyoto Kuno Hospital, Kyoto 605-0981, Japan; 9 Department of Physical Therapy, Faculty of Rehabilitation Science, Kobe International University, Kobe 658-0032, Japan; 10 MIZ Co., Ltd., Saga 840-0054, Japan; 11 BME Research Laboratory, Sosei Ltd., Hamamatsu 432-8002, Japan

**Keywords:** age-adjusted profiling, Color Kanji Pick-Out Test, community-dwelling older adults, executive function, MMSE ceiling effect, mobility, preventive physical therapy, cognitive–motor coupling

## Abstract

**Highlights:**

**What are the main findings?**

- Among community-dwelling older adults with near-ceiling MMSE scores, an age-adjusted Color Kanji Pick-Out Test (CKPT) app score (ΔINDEX1) identified two mobility indicators—Timed Up and Go (TUG) and maximum walking speed—that differed significantly between groups and remained statistically significant after Benjamini–Hochberg false discovery rate (FDR) correction across 70 consolidated indicators (q = 0.029 and q = 0.044, respectively).
- Additional uncorrected associations with single-leg stance time, step length, usual walking speed, sarcopenia, bodily pain, and body mass index did not survive FDR correction and should be regarded as exploratory.

**What are the implications of the main findings?**

- Age-adjusted executive functioning profiling using the CKPT app can detect mobility-related differences among older adults whose conventional cognitive screening results are uniformly normal, supporting its use as a feasible adjunct to community-based screening.
- Individuals with relatively lower age-adjusted CKPT app performance may be candidates for early multicomponent interventions that combine physical training, cognitive stimulation, and support for health behavior maintenance, although prospective validation is necessary.

**Abstract:**

**Background/Objectives:** The Mini-Mental State Examination (MMSE) often shows ceiling effects in community-dwelling older adults, limiting the detection of subtle functional vulnerability. We examined whether an age-adjusted score derived from the Color Kanji Pick-Out Test (CKPT) app could identify functional heterogeneity among older adults with near-ceiling MMSE scores. **Methods:** In this cross-sectional study, 155 community-dwelling older adults aged ≥65 years underwent CKPT app assessment. An age-adjusted score (ΔINDEX1) was calculated as the residual from a linear regression of INDEX1 on age, and participants were classified into two groups using a median split. Group differences in cognitive, physical, psychological, and lifestyle variables were examined across 70 indicators retained after consolidation (Spearman’s |r| ≥ 0.75). Effect sizes (rank-biserial r and Cramer’s V) were reported, and false discovery rate (FDR) correction was applied (Benjamini–Hochberg). **Results:** MMSE scores were uniformly high in both the ΔINDEX1 groups (median 29–30, *p* = 0.138). Of the 70 indicators, 10 reached uncorrected significance (*p* < 0.05). After FDR correction, both Timed Up and Go (TUG) time (*p* < 0.001, r = 0.33, q = 0.029) and maximum walking speed (*p* = 0.001, r = 0.30, q = 0.044) remained statistically significant. Other uncorrected associations (single-leg stance, step length, usual walking speed, sarcopenia, bodily pain, and BMI) did not survive FDR correction and should be regarded as exploratory. **Conclusions:** Age-adjusted CKPT app profiling was associated with mobility-related differences in TUG and maximum walking speed, both significant after FDR correction, despite uniformly high MMSE scores. Psychosocial and lifestyle associations were preliminary and require confirmation in future studies. Because ΔINDEX1 was both derived and tested within the same predominantly female sample (86.5% women), these cross-sectional findings require external validation before generalization, particularly in older men.

## 1. Introduction

The rapid growth of the older adult population worldwide [1] has intensified the importance of healthy aging, a concept centered on maintaining functional ability and intrinsic capacity in later life [2,3]. Although dementia prevention has attracted substantial attention [4], accumulating evidence suggests that subtle deterioration in cognitive, physical, and psychosocial functions may begin years before overt clinical impairment becomes apparent [5,6]. Accordingly, there is a growing need for community-based screening tools capable of detecting these early pre-diagnostic changes and supporting timely preventive intervention.

The Mini-Mental State Examination (MMSE) is one of the most widely used cognitive screening instruments in clinical and community settings [7]. However, its utility in cognitively intact, community-dwelling older adults is substantially limited by a well-recognized ceiling effect [8,9]. In practice, many older adults without dementia score near the upper end of the MMSE, leaving little room to detect subtle individual differences in cognitive function. In addition, cognitive performance is inherently influenced by age [5]. As a result, identical raw cognitive scores may reflect different functional meanings in individuals of different ages, making it difficult to distinguish age-related changes from a relatively excessive decline. This represents a fundamental limitation of unadjusted cognitive screening when the goal is to identify preclinical vulnerability.

Executive functioning, including the ability to plan, monitor, and flexibly adapt goal-directed behavior, has been identified as a particularly sensitive domain for detecting early functional decline in older adults [5,10]. Executive functioning is closely linked to physical performance, including gait and balance, with prefrontal involvement in attention-demanding locomotion repeatedly demonstrated [11,12]. This cognitive–motor coupling suggests that assessment tools capable of capturing executive functioning under ecologically relevant dual-task conditions [13] may provide insight not only into cognition itself but also into integrated functional status, including what the World Health Organization defines as intrinsic capacity [3].

The Color Kanji Pick-Out Test (CKPT) app is a tablet-based assessment tool designed to evaluate executive functioning and working memory under dual-task conditions that simulate the cognitive demands of everyday activities [14]. To date, however, the CKPT app has been validated primarily by our research group, and independent replication in cohorts assessed by other investigators remains limited. While the present study was conducted within a single research program, it begins to address this gap by extending the approach to a new community sample. Unlike conventional screening approaches that yield coarse categorical results, the CKPT app provides a continuous composite performance score (INDEX1), allowing for finer characterization of individual variation. However, as with other cognitive measures, raw INDEX1 values are affected by age-related variance, which may obscure clinically meaningful differences between individuals and groups. To address this issue, we derived an age-adjusted residual score (ΔINDEX1) by regressing INDEX1 on age, thereby isolating the performance component that is independent of chronological aging.

From a technological standpoint, the CKPT app represents a tablet-based assessment platform that integrates automated task delivery, real-time response logging, and instant composite scoring without requiring specialized staff or the paper-based administration of the test. These features align with the growing application of digital health and telerehabilitation technologies in rehabilitation and health promotion settings [15,16]. The age-adjusted residual scoring approach used in this study, whereby individual performance is benchmarked against an age-expected trajectory derived from population-level regression, is conceptually compatible with digital biomarker-based risk stratification [17,18], although the present study did not implement or validate predictive or AI-based models. By placing the CKPT app within the context of digital health and telerehabilitation technologies, this study contributes to the clinical application of automated cognitive–motor assessment in preventive physical therapy.

This cross-sectional study aimed to examine whether ΔINDEX1 could identify functional heterogeneity among community-dwelling older adults with near-ceiling MMSE scores. Specifically, we investigated whether relatively lower age-adjusted executive functioning was associated with differences in physical performance, psychological characteristics, and lifestyle-related factors. We hypothesized that participants in the low-performance group (Low group) would demonstrate poorer physical function than those in the high-performance group, and we anticipated that mobility-related measures would be the most sensitive, because attention-demanding locomotion shares prefrontal executive and attentional resources with the CKPT task, so that early executive decline would manifest preferentially in dual-task-like motor performance rather than in static or strength-based measures, despite similarly high MMSE scores, suggesting that age-adjusted CKPT app profiling may capture early integrated functional vulnerability that conventional cognitive screening fails to detect.

## 2. Materials and Methods

### 2.1. Study Design and Participants

This cross-sectional observational study included community-dwelling older adults aged ≥65 years who were recruited from physical fitness assessment events held in Imari City (Saga Prefecture) and Koka City (Shiga Prefecture), Japan. The events were conducted annually between August and September from 2021 to 2025. The eligibility criteria included the ability to walk independently and provide written informed consent. Individuals diagnosed with dementia or severe psychiatric disorders were excluded. Of the 805 older adults assessed at the six contributing events (Imari 2021, 2023, 2024, and 2025; Koka 2023 and 2024), those who were free of dementia, met the eligibility criteria, and provided consent for and completed the CKPT app assessment with data on the core study variables yielded 163 participants. After excluding eight individuals with missing data on other key variables, 155 participants were included in the final analysis. The study was approved by the Ethics Committee of Kyoto Tachibana University (approval no. 18–26, date of approval: 18 July 2018), under which the ongoing community cohort study was originally registered and continues to operate and was conducted in accordance with the Declaration of Helsinki.

### 2.2. Relationship to Prior Publication and Sample Overlap

This study used data from the ongoing community cohort described by Goda et al. [19]. To clarify the distinct contributions of the present analysis and address potential concerns regarding sample overlap, the relationship between the two studies is summarized in Table 1. In brief, Goda et al. [19] examined the association between raw CKPT app performance and cognitive frailty in women with MMSE ≥ 27 using regression-based adjustment for age, MMSE, and education, whereas the present analysis addresses a methodologically and conceptually distinct question: whether an age-adjusted residual score derived from the same instrument differentiates physical, psychosocial, and lifestyle profiles in a broader, sex-inclusive sample (both men and women) that incorporates an additional year of follow-up data (2025). Although a substantial proportion of participants is common to both datasets, the outcome of interest, analytic framework, and conclusions drawn are nonoverlapping.

### 2.3. Measures

#### 2.3.1. Cognitive Assessment

The Mini-Mental State Examination (MMSE) [7] was used as a conventional cognitive screening tool. Executive functioning and dual-task performance were assessed using the Color Kanji Pick-Out Test (CKPT) app, a tablet-based application developed for the assessment of early-stage cognitive decline [14]. The CKPT app requires participants to silently read a short story displayed on a 12.9-inch iPad Pro while simultaneously performing a Stroop-like color-kanji judgment task: participants tap once when the meaning and font color of a color-kanji character match and twice when they do not. Responses were recorded over 2 min, followed by three multiple-choice recognition questions about the story content. The test was administered via automated voice guidance and scoring, enabling a standardized assessment without the need for specialized staff. By requiring participants to maintain and comprehend a narrative while concurrently performing a Stroop-like color-kanji judgment, the CKPT app engages several interrelated cognitive domains rather than a single isolated function. The Stroop-like component imposes demands on inhibitory control and selective attention, as participants must suppress the automatic reading of the kanji meaning to judge the font color. The dual-task structure—reading comprehension performed concurrently with color judgment—additionally taxes the working memory and attentional switching required to coordinate two competing streams of processing. Subsequent recognition questions probe episodic encoding and retrieval of narrative content. Performance on the task therefore reflects predominantly executive and attentional processes—particularly inhibitory control, working memory, and attentional shifting—that are known to decline early in the trajectory of cognitive aging and are not well captured by global screening instruments such as the MMSE. The primary outcome, INDEX1, was a continuous composite score calculated as the number of correct color-judgment responses multiplied by the proportion of correct recognition answers, with higher scores indicating better performance. In the present sample, the observed INDEX1 values ranged from approximately 0 to 25, with a median of approximately 9. The CKPT app has demonstrated acceptable test–retest reliability and criterion validity, with significant correlations with conventional executive functioning measures, including the Wisconsin Card Sorting Test [14,20]. In a prior study using this cohort, CKPT app performance was independently associated with cognitive frailty after adjusting for MMSE score, age, and education [19].

#### 2.3.2. Physical Performance

Physical function was assessed using the Timed Up and Go test (TUG) [21], maximum walking speed, usual walking speed, maximum step length, usual step length [22], and single-leg standing time (SLST). Sarcopenia status was classified according to the Asian Working Group for Sarcopenia 2019 criteria [23], and frailty phenotype was categorized using the Japan Cardiovascular Health Study (JCHS) criteria [24].

#### 2.3.3. Psychological and Lifestyle Measures

Psychological and lifestyle-related variables were assessed using a structured questionnaire. These included bodily pain, boredom, and participation in regular exercise. Health-related quality of life was assessed using the EuroQol 5-Dimension (EQ-5D) [25]. Subjective cognitive decline (SCD) [26] was operationally assessed using the cognitive domain of the Kihon Checklist (KCL-CF), an instrument with established validity for frailty screening that has been employed in prior Japanese cognitive frailty research [19,27], comprising three self-reported items concerning memory complaints, telephone number lookup behavior, and date orientation. Each item was scored as 0 or 1, and a total score of ≥1 (range 0–3) was considered indicative of SCD.

### 2.4. Derivation of the Age-Adjusted CKPT App Score (ΔINDEX1)

In preliminary analyses of this dataset, profiling based on the CKPT app was explored in relation to Motoric Cognitive Risk (MCR) [28]; however, cluster-based approaches did not yield clinically meaningful group separations. During these analyses, a significant negative association was observed between age and INDEX1 (Spearman’s r = −0.382, *p* < 0.001), suggesting that age-related variance in raw CKPT app performance may obscure meaningful differences between individuals. Accordingly, as a secondary data-driven exploratory analysis, we derived an age-adjusted score to examine whether relative performance—independent of chronological age—would more sensitively differentiate functional profiles within this sample.

ΔINDEX1 was defined as the residual from a simple linear regression of INDEX1 on age. Prior to model fitting, the relationship between age and INDEX1 was visually inspected and quantitatively assessed. A LOWESS curve overlay showed an approximately linear trend, and a quadratic term for age, when added to the model, was non-significant (*p* > 0.10), supporting the assumption of linearity. No substantial evidence of influential outliers (Cook’s distance) was identified. The regression equation is as follows:

INDEX1 = −0.2613 × age + 28.5734

A positive ΔINDEX1 indicated that a participant performed better than expected for their age, whereas a negative ΔINDEX1 indicated relative underperformance compared to age-matched peers. Participants were classified into two groups using a median split of ΔINDEX1: the Low group (≤median) and the High group (>median). Because no established clinical cutoff exists for this novel index, a median-based approach was adopted to avoid arbitrary threshold selections. We acknowledge that dichotomization reduces statistical power and can introduce an arbitrary boundary [29]; the potential implications of this dichotomization for statistical inference are addressed in the Limitations section.

### 2.5. Statistical Analysis

The participant characteristics between the ΔINDEX1 groups are summarized as median and interquartile range (IQR) for continuous variables and frequency and percentage for categorical variables. Between-group differences were assessed using the Mann–Whitney U test for continuous variables and the chi-square test or Fisher’s exact test, as appropriate, for categorical variables.

To reduce redundancy among highly correlated variables, a preliminary Spearman correlation analysis was conducted, and variables with |r| ≥ 0.75 within conceptually similar domains were consolidated into representative measures. This consolidation procedure was pre-specified before group comparisons were performed and yielded a final set of 70 indicators that were carried forward for inferential analysis. The complete set of 70 indicators, including group statistics, *p*-values, effect sizes, and FDR-adjusted q-values, is listed in Table S1. Effect sizes were calculated for all comparisons: rank-biserial correlation (r) for Mann–Whitney U tests [30] and Cramer’s V (V) for chi-square tests.

Because this study had an exploratory aim, no formal correction for multiple comparisons was applied as the primary analysis strategy. As a supplementary analysis, all *p*-values across the 70 consolidated indicators were subjected to false discovery rate (FDR) correction using the Benjamini–Hochberg procedure [31]. We further note that ΔINDEX1 was both derived from and tested within the same sample; this internal use of a regression equation in subsequent group comparisons may inflate the apparent strength of associations, and external validation in an independent reference sample with pre-specified regression coefficients is required before the metric can be generalized (see Section 4.6 (Limitations)).

As a confirmatory sensitivity analysis addressing the limitations of dichotomization, ΔINDEX1 was additionally modeled as a continuous predictor (per 1-SD increment) in multivariable models adjusted for sex and education—linear regression for the two mobility outcomes that survived FDR correction (TUG and maximum walking speed) and logistic regression for sarcopenia. Age was not included as a covariate because ΔINDEX1 is the age-residualized component of INDEX1. Robustness to non-normal residuals was confirmed using heteroscedasticity-consistent (HC3) standard errors (SEs).

All analyses were performed using Python 3.11 (Python Software Foundation, Wilmington, DE, USA) with SciPy version 1.13.0, and statistical significance was defined as a two-sided *p*-value of < 0.05. The analysis code is available from the corresponding author upon reasonable request.

## 3. Results

### 3.1. Participant Characteristics

A total of 155 community-dwelling older adults were included in the final analysis (median age 75.0 years, IQR 69.5–78.0; 134/155 [86.5%] women). Age and sex did not differ significantly between the ΔINDEX1 groups (*p* = 0.224 and *p* = 1.000, respectively), indicating that group assignment was not driven by demographic imbalances. The participant characteristics and outcomes of the ΔINDEX1 group are summarized in Table 2.

### 3.2. MMSE Scores Between ΔINDEX1 Groups

MMSE scores were uniformly high between the two groups, with a median of 29–30 points in each group and no statistically significant difference between the groups (*p* = 0.138). Both groups showed a near-ceiling distribution, with interquartile ranges confined to 29–30 participants. In addition, ΔINDEX1 was not significantly correlated with MMSE (Spearman’s r = 0.090, *p* = 0.265), suggesting that the two measures captured different aspects of cognitive function and that group assignment was not confounded by gross cognitive status.

### 3.3. Physical Performance Between ΔINDEX1 Groups

Despite the absence of MMSE differences, multiple physical performance measures differed significantly between the ΔINDEX1 groups. Two indicators remained statistically significant after the FDR correction among the 70 consolidated indicators. TUG time showed the strongest association (*p* < 0.001, r = 0.33, q = 0.029), with longer times in the Low group (median 6.05 s, IQR 5.63–6.74) than in the High group (median 5.60 s, IQR 5.00–6.24). The maximum walking speed was also significantly slower in the Low group (median 175.8 cm/s) than in the High group (median 191.1 cm/s; *p* = 0.001, r = 0.30, q = 0.044). Additional gait- and balance-related indicators differed at the uncorrected level in the same direction: maximum step length (*p* = 0.005, r = 0.26), single-leg stance time (*p* = 0.006, r = 0.26), usual walking speed (*p* = 0.007, r = 0.25), usual step length (*p* = 0.007, r = 0.25), and sarcopenia criteria (*p* = 0.005, V = 0.23); however, these did not survive FDR correction and should be interpreted as exploratory findings. The effect sizes were small to moderate (r range: 0.22–0.33), consistent with the subtle functional differences expected in a cognitively intact community-based sample.

### 3.4. Psychological and Lifestyle Correlates of ΔINDEX1

Bodily pain was reported more frequently in the Low group (70.5%) than in the High group (50.6%; *p* = 0.018, V = 0.19) at the uncorrected level. The body mass index (BMI) was also higher in the Low group at the uncorrected level (median 23.30 vs. 22.00 kg/m^2^; *p* = 0.016, r = 0.22). Neither of these associations survived the FDR correction and should, therefore, be interpreted as preliminary signals warranting further investigation.

### 3.5. Subjective Cognitive and Quality-of-Life Measures

The prevalence of subjective cognitive decline tended to be higher in the Low group (36.8%) than in the High group (23.7%); however, the difference was not statistically significant (*p* = 0.071, V = 0.15). The EQ-5D scores also did not differ significantly between the groups (*p* = 0.340, r = 0.08). Thus, ΔINDEX1 was not significantly associated with subjective cognitive complaints or health-related quality of life in this population.

### 3.6. Sensitivity Analysis: ΔINDEX1 as a Continuous Predictor

When ΔINDEX1 was modeled as a continuous predictor adjusted for sex and education, the two mobility associations that survived the FDR correction remained statistically significant. Each 1-SD increase in ΔINDEX1 was associated with a shorter TUG time (β = −0.19 s; 95% CI, −0.35 to −0.04; *p* = 0.015; *n* = 154) and a faster maximum walking speed (β = 6.20 cm/s; 95% CI, 1.36 to 11.04; *p* = 0.012; *n* = 153); the results were materially unchanged using HC3 robust standard errors (*p* = 0.018 and *p* = 0.011, respectively). In contrast, the association with sarcopenia was attenuated and non-significant in the adjusted continuous model (odds ratio per 1-SD = 0.77; 95% CI, 0.48–1.24; *p* = 0.280; *n* = 152), consistent with its exploratory status. These results indicate that the principal mobility findings were not artifacts of the median-split dichotomization and were independent of sex and educational level.

## 4. Discussion

### 4.1. Principal Findings

This cross-sectional study examined whether an age-adjusted CKPT app score (ΔINDEX1) could differentiate functional profiles among community-dwelling older adults who were cognitively intact according to the conventional MMSE screening. The principal finding was that participants with relatively lower age-adjusted executive function showed poorer mobility, specifically in TUG performance and maximum walking speed, both of which remained statistically significant after FDR correction (q = 0.029 and q = 0.044, respectively), despite uniformly near-ceiling MMSE scores. Additional associations with single-leg stance time, step length, usual walking speed, sarcopenia, bodily pain, and BMI were observed at the uncorrected level only, did not survive the FDR correction, and should be regarded as exploratory. These findings suggest that age-adjusted executive functioning profiling using the CKPT app can detect mobility-related differences that conventional cognitive screening does not, whereas associations with psychosocial and lifestyle factors require confirmation.

### 4.2. Overcoming the MMSE Ceiling Effect Through Age Adjustment

The ceiling effect of the MMSE in community-dwelling older adults is a well-recognized limitation of conventional cognitive screening tests [8,9]. In the present sample, the interquartile range of MMSE scores was confined to 29–30 points in both ΔINDEX1 groups, illustrating the limited discriminative capacity of the MMSE in a highly functioning population. In addition, ΔINDEX1 was not significantly correlated with the MMSE, indicating that the two measures captured different aspects of cognitive function.

The rationale for age adjustment lies in the significant negative association between age and the raw INDEX1. By isolating the residual from this age-related trajectory, ΔINDEX1 shifts the interpretation from absolute to age-relative performance, addressing the clinically relevant question of whether an individual’s executive function falls below what would be expected for their age. This reframing may be particularly useful in highly functioning older populations, for whom conventional global cognitive screening lacks sensitivity. Instruments such as the Montreal Cognitive Assessment (MoCA) were developed partly to overcome the ceiling effect of the MMSE and to show greater sensitivity to executive and attentional decline. ΔINDEX1 is not intended to replace such instruments but to complement them; it is delivered automatically on a tablet without trained staff, yields a continuous rather than categorical score, and—because it is age-residualized—expresses performance relative to age-based expectation rather than against a fixed cut-off. These features make age-adjusted CKPT profiling well-suited for large-scale community screening, where administering a clinician-scored test, such as the MoCA, to all attendees would be impractical. A head-to-head comparison of ΔINDEX1 with MoCA in the same cohort is a logical next step.

The residual-based approach used here is complementary to, but distinct from, covariate adjustment for age in the multivariable models. Whereas multivariable adjustment removes the average linear effect of age while estimating associations with other variables, the residual ΔINDEX1 yields an explicit, individual-level index of age-relative executive performance that can be examined, ranked, or dichotomized—a property that aligns directly with the screening-oriented aim of identifying individuals who perform below age expectation. Therefore, these two strategies are not in conflict with each other. The present study and our earlier covariate-adjusted analysis [19] address related but different questions, and the confirmatory continuous model reported here (Section 3.6), in which ΔINDEX1 remained significant after adjustment for sex and education, indicates that the residual metric behaves consistently within a multivariable framework.

### 4.3. ΔINDEX1 as a Potential Indicator of Early Mobility Vulnerability

Two mobility indicators, TUG performance and maximum walking speed, were significantly associated with lower ΔINDEX1, and both associations remained significant after FDR correction. Participants in the Low group showed a median TUG time approximately 0.45 s longer than those in the High group (6.05 vs. 5.60 s) and a median maximum walking speed approximately 15.3 cm/s slower (175.8 vs. 191.1 cm/s).

To place these magnitudes in context, the between-group difference in TUG was approximately 0.45 s, which is below the published measurement-error thresholds for within-person change in community-dwelling older adults, including a smallest real difference of 1.1 s and a minimal detectable change of 2.08 s reported in previous studies [32,33]. Thus, the TUG findings should not be interpreted as indicating overt, clinically detectable impairment at the individual level; rather, they suggest a small but statistically robust group-level shift consistent with early functional variation. In contrast, the between-group difference in maximum walking speed was approximately 0.15 m/s, which approached the smallest real difference reported for the fastest gait speed in community-dwelling older adults (0.17 m/s) [32] and was comparable to or greater than the substantial meaningful-change benchmark of approximately 0.10 m/s proposed for usual gait speed [34]. Although these benchmarks were derived primarily for within-person change and the Perera benchmark refers to usual rather than maximum gait speed, the present findings suggest that the observed gait speed difference may have functional relevance at the group level. Taken together with the modest effect sizes (r ≈ 0.30–0.33), these results are best interpreted as subtle but potentially meaningful group-level differences that may reflect early mobility variation rather than established clinical impairments.

As noted, the clinical significance of these difference magnitudes warrants further calibration. Previous studies have shown that poorer TUG performance and slower gait speed are associated with fall risk and incident disability in community-dwelling older adults [35,36]. These differences emerged in a sample whose MMSE scores remained essentially indistinguishable, suggesting that age-adjusted executive functioning profiling can detect mobility-related differences that conventional global cognitive screening does not. The pattern was concordant across other gait- and balance-related indicators (usual walking speed, step length, and single-leg stance time), although these did not survive FDR correction and required independent confirmation. A plausible neurobiological basis for the observed coupling between executive functioning and mobility lies in the shared frontal and fronto-subcortical networks that subserve both domains. Attention-demanding locomotion, such as the rapid stand–turn–walk sequence of the TUG and walking at maximal speed, recruits prefrontal regions for goal maintenance, inhibitory control, and online allocation of attention, the same processes engaged by the CKPT task [11,12]. With advancing age, microstructural degradation of frontal white-matter tracts and the consequent disconnection of fronto-parietal and fronto-subcortical circuits are thought to weaken cognitive–motor integration so that subtle executive decline and subtle mobility decline may emerge in parallel before either reaches a clinically overt threshold [13]. Within this framework, ΔINDEX1 may index the integrity of these shared networks, which would help explain why an executive-loaded cognitive measure tracked mobility differences even when global cognition, as assessed by the MMSE, did not. At the molecular level, dysregulated nutrient-sensing pathways, such as mechanistic target of rapamycin (mTOR) signaling, have been implicated in both neuronal and musculoskeletal aging and may constitute a shared upstream substrate linking cognitive and motor decline, although the present study was not designed to address such mechanisms [37].

This interpretation is consistent with the established concept of cognitive–motor coupling [11,13]. Executive functioning and gait share overlapping neural substrates, with prefrontal involvement during attention-demanding locomotion repeatedly demonstrated [12], and mild cognitive impairment has been associated with measurable deterioration in gait and balance [38]. Because the CKPT app was designed to evaluate performance under dual-task conditions that resemble everyday cognitive demands, it may be particularly sensitive to early cognitive–motor vulnerabilities. The Low group also tended to include a higher proportion of participants meeting the sarcopenia criteria, which is broadly consistent with prior evidence linking sarcopenia and cognitive impairment [39], although this finding requires confirmation in adequately powered prospective studies.

### 4.4. Preliminary Psychosocial and Lifestyle Correlates

Beyond physical performance, lower ΔINDEX1 was associated at the uncorrected level with more frequent reports of bodily pain and higher body mass index (BMI). Bodily pain may represent a relevant pathway, as chronic pain can consume attentional and executive resources, thereby adversely affecting cognitive task performance [40]. However, because neither of these associations survived the FDR correction, any extension of the ΔINDEX1 profile beyond mobility-related domains should be regarded as a preliminary hypothesis to be tested rather than an established finding. These uncorrected patterns are compatible with the WHO framework of intrinsic capacity [3]; however, confirmation in independent samples is required before this interpretation can be confidently advanced.

The absence of a significant difference in subjective cognitive decline, in contrast to the objective mobility findings, suggests that ΔINDEX1 captures performance-based functional variation that older adults may not necessarily perceive or report on. This dissociation is consistent with the limited sensitivity of the brief three-item KCL-CF used here (see Limitations) and the well-documented imperfect correspondence between subjective complaints and objective performance in cognitively intact older adults.

### 4.5. Implications for Community-Based Screening and Preventive Intervention

The present findings suggest that ΔINDEX1, when interpreted with appropriate caution and pending validation of its scoring in an independent reference sample, may have practical value for community-based risk stratification, particularly for identifying older adults with normal MMSE scores who show subtle differences in mobility. Because the CKPT app can be administered quickly using a tablet-based platform, age-adjusted profiling may offer a feasible means of identifying individuals who already show subtle mobility differences in cross-sectional assessments.

From a preventive perspective, individuals in the low-performance group may be appropriate candidates for early multicomponent interventions that combine physical training, cognitive stimulation, and support for healthy behavior maintenance [41]. Simultaneously, the modest effect sizes and the fact that only two associations survived FDR correction indicate that ΔINDEX1 should not be used as a stand-alone determinant of risk. These practical implications are also derived from a sample that was predominantly female and recruited from voluntary health promotion events, characteristics likely to reflect a relatively health-conscious subgroup of community-dwelling older adults. Therefore, generalization should be approached with considerable caution.

From the perspective of digital health and telerehabilitation technologies [15,16], the CKPT app offers automated administration, objective response logging, and instant score calculation, thereby reducing the assessor’s burden and minimizing the potential for human error. The age-adjusted residual scoring approach introduced in this study accounts for an individual’s expected performance trajectory based on their chronological age. Although this approach is conceptually compatible with digital biomarker-based risk stratification [17,18], the present study did not implement or validate predictive or AI-based models, and any such application would require longitudinal data and prospective validation in independent cohorts.

Two related factors warrant consideration as potential confounders in this study. Education tended to be higher in the High group (*p* = 0.059), and because the CKPT app requires kanji reading and tablet operation, both formal education and digital literacy could plausibly influence app performance, independently of executive function. To address this, the confirmatory continuous analysis adjusted for education (Section 3.6) and the principal mobility associations with TUG and maximum walking speed persisted. Nonetheless, digital literacy was not directly measured and could not be fully controlled; therefore, residual confounding cannot be excluded from the study.

### 4.6. Limitations

This study has several limitations. First, the cross-sectional design precludes causal or temporal inference; it cannot be determined whether a lower ΔINDEX1 precedes physical decline or whether existing physical limitations adversely affect CKPT app performance. Second, although TUG and maximum walking speed remained significant after FDR correction across the 70 consolidated indicators (q = 0.029 and q = 0.044, respectively), the uncorrected psychosocial and lifestyle associations did not survive correction and should be regarded as exploratory rather than confirmed findings. Third, and most importantly, ΔINDEX1 was both derived from and tested within the same sample, which constitutes an internal statistical circularity that may inflate the apparent strength of the associations beyond what would be observed in an independent test set. Because no external healthy reference cohort was available, the regression coefficients reported here (INDEX1 = −0.2613 × age + 28.5734) are specific to the present sample and cannot be assumed to transfer to other populations; therefore, we regard this circularity as a major limitation of the study. Although a confirmatory continuous analysis adjusted for sex and education reproduced the principal mobility associations (Section 3.6), this internal robustness check does not substitute external validation. Accordingly, ΔINDEX1 should not be applied as a stand-alone screening or risk-stratification tool in other populations; future studies should pre-specify the regression equation using an independent reference sample or adopt cross-validation approaches before the residual metric is used for hypothesis testing or individual-level stratification. Fourth, the dichotomization of ΔINDEX1 via median split reduces statistical power and may introduce an arbitrary boundary [29]; analyses treating ΔINDEX1 as a continuous variable should be considered the methodologically preferred form in future replications. Fifth, the sample was predominantly female (86.5%), which limits generalizability to older adult males and other demographic groups; the small number of men precluded meaningful sex-stratified analyses, and the present associations should therefore be regarded as applying predominantly to community-dwelling older women. Moreover, because participants were recruited from voluntary health promotion events, the sample probably represents a relatively healthy, active, and health-conscious subgroup of the older population, introducing a selection bias that further limits external validity. Sixth, medication data were not available, and potentially important confounders, such as polypharmacy, sedative use, and analgesic use, could not be addressed. Seventh, the assessment of subjective cognitive decline relied on the three-item cognitive domain of the Kihon Checklist (KCL-CF); while the KCL-CF has demonstrated acceptable validity and reliability as a screening tool [27], its brevity may not fully capture the broader SCD construct [26], which could partly explain the absence of statistically significant differences between the groups. Finally, because the CKPT app requires kanji reading comprehension and basic tablet familiarity, its applicability to populations with varying literacy levels, digital skills, or different cultural settings remains to be investigated. Reassuringly, a confirmatory analysis modeling ΔINDEX1 as a continuous predictor adjusted for sex and education reproduced the significant associations with TUG and maximum walking speed (Section 3.6), indicating that the principal mobility findings were not driven by dichotomization.

## 5. Conclusions

This cross-sectional study provided preliminary evidence that an age-adjusted CKPT app score (ΔINDEX1) is associated with mobility-related functional differences, specifically in TUG performance and maximum walking speed, both of which remained statistically significant after FDR correction among community-dwelling older adults with near-ceiling MMSE scores. Additional associations with other physical, psychosocial, and lifestyle indicators were observed at the uncorrected level but did not survive correction for multiple tests and should be regarded as exploratory findings.

When interpreted using age-adjusted residual scoring, the Color Kanji Pick-Out Test (CKPT) app may identify subtle differences in mobility among older adults whose conventional cognitive screening results are within the normal range. This approach may help community-based screening efforts identify older adults who could benefit from further assessment, although these implications require confirmation through prospective studies with larger and more diverse sample sizes.

These conclusions should be interpreted in light of the cross-sectional nature of the study, the predominantly female sample, the internal derivation of the regression equation used to compute ΔINDEX1, and the absence of longitudinal follow-up and external validation. Prospective studies in larger, more diverse cohorts—preferably with externally derived regression coefficients and analyses treating ΔINDEX1 as a continuous variable—are needed to determine whether ΔINDEX1 can predict subsequent functional decline and to establish its clinical utility more definitively.

## Figures and Tables

**Table 1:** Comparison of the present study with the prior publication on the same community cohort.

Aspect	Goda et al. (Geriatrics, 2026) [19]	Present Study
Research question	Association between CKPT app performance and cognitive frailty	Whether age-adjusted CKPT app residual (ΔINDEX1) differentiates functional profiles among near-ceiling MMSE older adults
Sample	Subset of the same community cohort; women only; MMSE ≥ 27	Full cohort with one additional year of data (2025); both sexes; *n* = 155
Sample overlap	Reference cohort	Substantial overlap with the 2026 cohort plus newly recruited participants from the 2025 wave
Primary metric	Raw CKPT app INDEX1 + cognitive frailty classification	Age-adjusted residual ΔINDEX1 from linear regression of INDEX1 on age
Analytic approach	Multivariable regression with MMSE/age/education adjustment	Median-split group comparison across 70 consolidated indicators with FDR correction
Outcome focus	Cognitive frailty (categorical)	Physical, psychosocial, and lifestyle profiles (continuous and categorical)

CKPT, Color Kanji Pick-Out Test; MMSE, Mini-Mental State Examination; FDR, false discovery rate; ΔINDEX1, age-adjusted residual of CKPT app INDEX1.

**Table 2:** Participant characteristics and outcomes by ΔINDEX1 group (*n* = 155).

Variable	Total (*n* = 155)	Low (*n* = 78)	High (*n* = 77)	*p*-Value	Effect Size
Age (years)	75.0 (69.5–78.0)	75.5 (70.0–79.0)	74.0 (69.0–77.0)	0.224	r = 0.10
Sex (% female)	86.5% (134/155)	85.9% (67/78)	87.0% (67/77)	1.000	V = 0.02
Education (years)	12.0 (12.0–12.75)	12.0 (12.0–12.0)	12.0 (12.0–14.0)	0.059	r = 0.16
MMSE (points)	30.0 (29.0–30.0)	29.0 (29.0–30.0)	30.0 (29.0–30.0)	0.138	r = 0.12
TUG (s) **	5.87 (5.20–6.55)	6.05 (5.63–6.74)	5.60 (5.00–6.24)	<0.001	r = 0.33
Max walking speed (cm/s) **	183.8 (162.1–202.7)	175.8 (156.5–191.6)	191.1 (171.9–210.0)	0.001	r = 0.30
Max step length (cm) *	66.46 (61.91–72.67)	65.07 (60.72–70.29)	68.75 (63.90–73.88)	0.005	r = 0.26
Usual walking speed (cm/s) *	131.88 (112.60–143.13)	120.31 (108.51–138.82)	135.68 (117.91–144.18)	0.007	r = 0.25
Usual step length (cm) *	61.09 (56.14–65.54)	59.47 (54.70–64.37)	62.35 (57.60–66.84)	0.007	r = 0.25
Single-leg stance (s) *	24.7 (11.0–51.5)	20.0 (8.0–39.0)	30.4 (13.3–78.5)	0.006	r = 0.26
Grip strength (kg)	23.2 (20.7–27.0)	23.0 (20.0–26.1)	23.6 (21.0–27.8)	0.079	r = 0.14
Sarcopenia (% meeting criteria) *	13.8% (21/152)	21.8% (17/78)	5.4% (4/74)	0.005	V = 0.23
Bodily pain (% reporting) *	60.7% (94/155)	70.5% (55/78)	50.6% (39/77)	0.018	V = 0.19
BMI (kg/m^2^) *	22.70 (20.42–24.60)	23.30 (21.10–25.08)	22.00 (20.00–24.02)	0.016	r = 0.22
SCD (% positive)	30.2% (46/152)	36.8% (28/76)	23.7% (18/76)	0.071	V = 0.15
EQ-5D (utility)	0.82 (0.81–1.00)	0.81 (0.81–1.00)	0.92 (0.81–1.00)	0.340	r = 0.08

Values are median (IQR) unless otherwise stated; * indicates *p* < 0.05 (uncorrected); ** indicates statistical significance after Benjamini–Hochberg FDR correction across the 70 consolidated indicators (q < 0.05). After FDR correction, both TUG (q = 0.029) and maximum walking speed (q = 0.044) remained significant; all other associations did not survive correction. Effect sizes are reported as rank-biserial r for continuous variables (Mann–Whitney U test) and Cramer’s V for categorical variables (chi-square test). Owing to item-level missingness, effective sample sizes were max walking speed (*n* = 154), max step length (*n* = 150), usual walking speed (*n* = 154), usual step length (*n* = 152), grip strength (*n* = 154), sarcopenia (*n* = 152), BMI (*n* = 154), SCD (*n* = 152), and EQ-5D (*n* = 154); all other variables had complete data (*n* = 155). TUG, Timed Up and Go; MMSE, Mini-Mental State Examination; SCD, subjective cognitive decline; BMI, body mass index; EQ-5D, EuroQol 5-Dimension; IQR, interquartile range.

## Data Availability

The datasets presented in this article are not readily available because the data are part of an ongoing study. Requests to access the datasets should be directed to the corresponding author.

## Supplementary Materials

The following supporting information can be downloaded at: https://www.mdpi.com/article/10.3390/healthcare14131868/s1. Table S1: Group comparison across all 70 consolidated indicators between ΔINDEX1 median-split groups with Benjamini–Hochberg false discovery rate-adjusted q-values.

## Abbreviations

The following abbreviations are used in this manuscript:

AWGS	Asian Working Group for Sarcopenia
BMI	Body Mass Index
CKPT	Color Kanji Pick-Out Test
EQ-5D	EuroQol 5-Dimension
FDR	False Discovery Rate
IQR	Interquartile Range
JCHS	Japan Cardiovascular Health Study
KCL-CF	Kihon Checklist—Cognitive Function domain
LOWESS	Locally Weighted Scatterplot Smoothing
MCR	Motoric Cognitive Risk
MMSE	Mini-Mental State Examination
SCD	Subjective Cognitive Decline
TUG	Timed Up and Go
WHO	World Health Organization
